# Does physical fitness affect cognitive functions differently across adulthood? An advantage of being older

**DOI:** 10.3389/fpsyg.2023.1134770

**Published:** 2023-06-16

**Authors:** Patrick D. Gajewski, Klaus Golka, Jan G. Hengstler, Thura Kadhum, Jan Digutsch, Erhan Genç, Edmund Wascher, Stephan Getzmann

**Affiliations:** ^1^Leibniz Research Centre for Working Environment and Human Factors (IfADo) at the Technical University of Dortmund, Dortmund, Germany; ^2^Clinic for Psychosomatic Rehabilitation, Mittelrhein-Klinik, Boppard - Bad Salzig, Boppard, Germany; ^3^Institute of Behavioral Science and Technology, University of St. Gallen, St. Gallen, Switzerland

**Keywords:** aging, neuropsychology, physical working capacity, verbal memory, working memory, Stroop interference, structural equation modeling, cardiorespiratory fitness

## Abstract

**Introduction:**

There is a large interindividual variability in cognitive functioning with increasing age due to biological and lifestyle factors. One of the most important lifestyle factors is the level of physical fitness (PF). The link between PF and brain activity is widely accepted but the specificity of cognitive functions affected by physical fitness across the adult lifespan is less understood. The present study aims to clarify whether PF is basically related to cognition and general intelligence in healthy adults, and whether higher levels of PF are associated with better performance in the same or different cognitive functions at different ages.

**Methods:**

A sample of 490 participants (20–70 years) was analyzed to examine this relationship. Later, the sample was split half into the young to middle-aged group (YM; 20–45 years; *n* = 254), and the middleaged to older group (MO; 46–70 years; *n* = 236). PF was measured by a quotient of maximum power in a bicycle ergometry test PWC-130 divided by body weight (W/kg), which was supported by a self-reported level of PF. Cognitive performance was evaluated by standardized neuropsychological test batteries.

**Results:**

Regression models showed a relationship between PF and general intelligence (*g*-factor) and its subcomponents extracted using structural equation modeling (SEM) in the entire sample. This association was moderated by age, which also moderated some specific cognitive domains such as attention, logical reasoning, and interference processing. After splitting the sample into two age groups, a significant relationship was found between cognitive status, as assessed by the Mini Mental State Examination (MMSE), and PF in both age groups. However, apart from cognitive failures in daily life (CFQ), no other association between PF and specific cognitive functions was found in the YM group. In contrast, several positive associations were observed in the MO group, such as with selective attention, verbal memory, working memory, logical reasoning, and interference processing.

**Discussion:**

These findings show that middle-aged to older adults benefit more from PF than younger to middle-aged adults. The results are discussed in terms of the neurobiological mechanisms underlying the cognitive effects of PF across the lifespan.

**Clinical trial registration:**

https://clinicaltrials.gov/ct2/show/NCT05155397, identifier NCT05155397.

## Introduction

Cognitive functions show a progressive decline beginning in the middle-age (Verhaeghen and Salthouse, 1997; Verhaeghen and Cerella, 2002; Salthouse, 2006; Nyberg et al., 2012; Hartshorne and Germine, 2015; Salthouse, 2015, for reviews). This decline may lead to mild cognitive impairment (MCI) and increase the risk for dementia in older age, as they are part of the same continuum (Scheltens et al., 2021). However, the individual rate of cognitive changes with age varies considerably, and the resulting variability of cognitive performance in older age is large (Lindenberger and von Oertzen, 2006; MacDonald et al., 2006). This variability depends on genetic, metabolic, and cardiovascular factors, but environmental and lifestyle factors are also critical for preventing cognitive decline and maintaining cognitive performance into old age (Hertzog et al., 2008; Bamidis et al., 2014; Beydoun et al., 2014; Parimon et al., 2014; Wu et al., 2014; Ballesteros et al., 2015). One of the most important lifestyle factors for mental health that is known to prevent cognitive impairment, dementia, and Alzheimer’s disease is regular physical activity (Laurin et al., 2001; Rovio et al., 2005; Hillman et al., 2008; Willey et al., 2016; Kivipelto et al., 2018; Erickson et al., 2019). Physical activity was broadly defined as “any bodily movement produced by skeletal muscles that require energy expenditure” (Denkinger et al., 2012; Iso-Markku et al., 2020). It increases oxygen uptake in the blood and has a positive effect on metabolism, immune system, and neuronal plasticity (Hillman et al., 2008; Erickson et al., 2011; Chapman et al., 2013; Bröde et al., 2022). Specifically, neuronal plasticity is thought to be triggered by the upregulation of neurotrophins, which promote neurogenesis and synaptic plasticity in the hippocampus and frontal brain areas important for memory and executive functions (Gomez-Pinilla and Hillman, 2013; Bamidis et al., 2014; Brehmer et al., 2014; Stillman et al., 2020).

Physical fitness (PF) can be improved through regular physical activity, which can have different types, facets, and formats (Iso-Markku et al., 2020). The main types of physical exercise are aerobic training (e.g., Hindin and Zelinski, 2012), strength/resistance training (e.g., Busse et al., 2009; Liu-Ambrose, 2010), and balance/coordination training (e.g., Voelcker-Rehage et al., 2011) or a combination of all of them (Barha et al., 2017). Apart from targeted physical activity, a general PF level is determined by a spectrum of daily activities and ranges from a sedentary lifestyle with only necessary activity to high-level habitual physical activity. In epidemiological studies that do not explicitly focus on a specific type of physical activity, a wide range of PF levels is common (Wu et al., 2014). PF is often measured through self-report, whereas objective measures such as VO_2_ maximum test, i.e., maximal oxygen consumption (Myers et al., 2002), or bicycle ergometry using an electrocardiogram (Campbell et al., 2001) seem to be more reliable and valid. Thus, supplementing self-report with an objective measurement could increase the validity of participants’ PF levels.

There are several studies reporting association between PF and cognition in healthy adults in observational or epidemiological settings (Etnier et al., 2006; Hillman et al., 2008; Busse et al., 2009; Voelcker-Rehage and Niemann, 2013; Kelly et al., 2014; Parimon et al., 2014; Ballesteros et al., 2015; Cox et al., 2016; Gajewski and Falkenstein, 2016, 2018; Barha et al., 2017, for reviews). Most of these studies have been conducted with older adults (65 years or older). For example, in the Nurses’ Health Study involving 18,766 older women, Weuve et al. (2004) examined the association between long-term moderate physical activity and cognitive function and found that higher levels of physical activity were associated with better cognitive performance and a lower risk of cognitive decline. In the review by Carvalho et al. (2014), 26 studies reported a positive correlation between physical activity and maintenance of cognitive function. This positive relationship was confirmed in further meta-analyses (Beydoun et al., 2014; Karr et al., 2014; Karssemeijer et al., 2017; Song et al., 2018).

Cross-sectional studies and randomized controlled trials reported consistent associations between PF and executive functions, e.g., inhibitory control (Forte et al., 2013; Nouchi et al., 2013; Berryman et al., 2014; Desjardins-Crépeau et al., 2014), Stroop interference (Liu-Ambrose, 2010; Gajewski and Falkenstein, 2015a; Coetsee and Terblanche, 2017), task switching (Hillman et al., 2006; Gajewski and Falkenstein, 2015b), or auditory distraction (Getzmann et al., 2013). Other authors found effects on episodic memory (Ruscheweyh et al., 2011; Nouchi et al., 2013), spatial memory (Erickson et al., 2011; Nagamatsu et al., 2013), or general cognition (Iso-Markku et al., 2016). Colcombe and Kramer (2003) reported in a meta-analysis of 18 randomized controlled studies the largest effects of PF on executive functions in adults being 55 years or older (see also Kramer and Colcombe, 2018). Erickson et al. (2019) showed in a recent review evidence from randomized controlled trials that indicates an association between moderate-to-vigorous intensity physical activity and improvements in processing speed, memory, and executive functions in adults older than 50 years. Likewise, in a comprehensive review of 42 randomized controlled trials with 3,781 healthy older adults Hindin and Zelinski (2012) stated that extended aerobic training has moderate to medium-sized effects on performance on untrained cognitive tasks for healthy adults older than 55 years. Other meta-analyses provided less consistent results: Smith et al. (2010) reported results of a meta-analysis in adults older than 18 years that showed modest improvements in processing speed, attention and executive functions after moderate physical training, whereas effects on working memory were less consistent. Similarly, Etnier et al. (2006) included in a meta-analysis all age group of adults (17–85 years) and did not observe an overall relationship between PF and cognitive functioning, but there was a different effect moderated by the age group. Additionally, there are some negative results of meta-analyses showing no, or only weak effects of PF on cognitive functions even after longer activity (e.g., Van Uffelen et al., 2008; Kelly et al., 2014; Sink et al., 2015; Diamond and Ling, 2016). This may be due to the large inhomogeneity of the studies, resulting from (a) inhomogeneous populations (clinical and non-clinical), small sample sizes, different age ranges, gender, education, etc., (b) differences in training regimes (type of activity, total duration, frequency and intensity, adherence), and (c) variability in data sets due to different cognitive measures. Only a few studies used comprehensive cognitive test batteries covering the most crucial functions (attention, memory, or executive functions) and their subfunctions (e.g., verbal, spatial, episodic, short-term memory, long-term memory) or executive functions (e.g., interference processing, inhibition, switching, updating, multitasking, working memory). Moreover, it is important to use standardized cognitive tests to enhance comparability between studies.

The findings presented above show a general relationship between PF and cognitive functions, but the results are inconsistent. The effects of PF on cognition may be attenuated when meta-analyses consider all age groups. Thus, little is known about possible changes in the relationship between PF and cognition across the adult lifespan, mainly in young to middle-aged adults. Indeed, most studies that showed positive effects or associations between PF and cognition were conducted with middle-aged to older adults (over 50 years), whereas studies with young to middle-aged adults are underrepresented in the literature (Voelcker-Rehage and Niemann, 2013; Erickson et al., 2019). This shows the need for a broad investigation of this relationship over the life span, to be able to map the development of this phenomenon. Moreover, it is not clear which specific cognitive functions are particularly associated with PF in young to middle-aged and middle-aged to older adults.

### The present study

The study is a part of the *Dortmund Vital Study* (Gajewski et al., 2022), an interdisciplinary cross-sectional and longitudinal study with up to three planned follow-up measures every 5 years, evaluating sources of inter-individual differences in cognitive functioning across the lifespan. The present cross-sectional part of the study used the baseline measures to analyze the relationship between the level of PF and a spectrum of cognitive functions in general population aged between 20 and 70 years. Additionally, this association was analyzed separately in younger to middle-aged and middle-aged to older adults to assess specific age-related differences in the relationship between PF and cognitive functions. Thus, the sample was dichotomized per median split in younger to middle-aged group (YM) and middle-aged to older group (MO) than the median age.^1^ Cognitive functions were measured by standardized psychometric tests covering the most important functions that decline with age such as the general cognitive status, psychomotor speed, selective attention and attentional endurance, verbal fluency, verbal short-term memory, working memory, interference processing, spatial cognition, logical reasoning, and cognitive failures in daily life. Age-related differences in these tests were reported previously (Gajewski et al., 2018). Additionally, we included in the analysis the *g*-factor assumed to reflect a proxy for general intelligence (Deary, 2020) that was determined using structural equation modeling (SEM). The aim of the study was to explore which specific cognitive functions are enhanced in individuals with high-level of PF and whether the role of PF level in cognition is the same or different in younger and older adults. In other words, the main question is whether the PF is universally related to particular cognitive functions regardless of age.

According to the literature, we expected a general association between PF and cognitive functions and general intelligence in a sample of healthy adults. To assess the modulation of the PF – cognition association by age, a moderator variable Age x PF was additionally included in the regression models. We hypothesized that PF would be associated with several cognitive functions, mainly executive functions in middle-aged and older adults, as previously reported. While the association of PF and cognitive functions should be well pronounced in the MO group, we expected that the relationship to be less consistent or even absent in the YM group, explaining the lack of corresponding reports and publication bias in the literature toward older age. We were particularly interested in responsivity of specific cognitive functions to PF as a function of age group.

## Materials and methods

### Study population

Participants of the *Dortmund Vital Study* (Clinicaltrials.gov: NCT05155397) are healthy adults aged between 20 and 70 years, with ages distributed almost evenly across the decades. The sample and further details like eligibility criteria, methods, and procedures are described in detail in the study protocol (Gajewski et al., 2022). Four hundred and ninety participants (aged 20–70 years, *M* = 43.9, SD = 13.9, 61% female) completed a comprehensive neuropsychological test battery and the bicycle ergometry during the baseline measurement. The study was approved by the IfADo Ethics Committee in October 2015 (A93-1). Data were collected between 2016 and 2020. Currently, the first follow-up data are collected. Thus, longitudinal data will be available in 2026 at the latest.

### Measurement of physical fitness

Participants’ current physical performance level was measured using a bicycle ergometer with a physical work capacity (PWC-130) cycle test. The PWC-130 is a version of the PWC-150 test adapted for older adults (Campbell et al., 2001). Because younger, middle-aged, and older individuals will participate in up to three follow-up tests in the present study, the protocol is a safe alternative to PWC-150 for all participants. This test aims to predict the absolute power output at a projected heart rate of 130 beats per minute. Relative power output is calculated by the power-to-weight ratio. In addition, pulse, electrocardiography (ECG) during rest, and systolic and diastolic blood pressure before and during ergometry are measured. Additionally, height, weight, waist-to-hip ratio, and Body Mass Index (BMI) are obtained from each participant.

Subjective assessment of PF in the last two years was provided using an adapted version of the Lüdenscheid Physical Activity Questionnaire (LPAQ) (Lüdenscheid Aktivitätsfragebogen, Höltke and Jakob, 2002). The questionnaire is aimed at assessing PF and weekly energy metabolism due to sport activities as indices for risk factors for cardiovascular diseases. The questionnaire consists of 13 questions, such as, “How long do you walk per week?,” “How often do you climb stairs?,” “How long do you cycle per week?,” “How frequently did you exercise which type of sport in the last 2 years?” or “How much time do you spend on physical exercise per week?.” The self-reported physical activity assessed by the LPAQ (in minutes per week) satisfactorily correlated with the maximal power of the PWC-130 test (*r* = 0.284, *p* < 0.001).

### Neuropsychological testing

A wide range of cognitive functions was evaluated using standardized neuropsychological tests, measuring general cognitive status, memory span and working memory, semantic memory in written and oral version, selective attention and attentional endurance, different aspects of verbal memory like learning and memory retrieval, psychomotor performance and speed of processing, interference control and inhibition, task switching, logical reasoning and spatial rotation. In the following the main characteristics of the tests are described. More specific details of the psychometric tests are described in Gajewski et al. (2018). Dependent variables used in the analyses are indicated in *italics*.

### MMSE

Mini-Mental-State-Examination (MMSE; Folstein et al., 1975) measures general cognitive status and is used for detecting early signs of cognitive impairment. The total score (maximum 30) was used as a dependent variable.

### D2 test

The attentional endurance test d2-R (*d2*; Brickenkamp, 2002) requires a fast search for stimuli according to predefined visual features. It consists of 14 lines, each composed of 47 characters (the letters “d” and “p” with one to four dashes (‘) above and/or below each letter). The subjects have 20 s per line to cross out the target stimuli “d” with two dashes. The total number of correctly identified d’s with two dashes represents the test score. The *d2* test is a measure of focused and sustained attention as well as processing speed. The total number of correctly identified symbols was used as a dependent variable.

### MWT-B

The Multiple-Choice Vocabulary Test (MWT-B; Lehrl, 1995) assesses word knowledge and thus aspects of crystalline intelligence. The test consists of 37 items with five words each. Only one of the five words reflects a meaningful word, the other similar words are meaningless. The subjects were required to mark the correct word. The difficulty of items increased with an increasing item number. The number of correctly identified meaningful words allows for assessment of the crystalline intelligence.

### LPS

The performance testing system (LPS; Horn, 1983) consists of 14 subtests and measures both aspects of fluid and crystallized intelligence. It has been normed on a large sample and is a reliable and valid measurement of intelligence. Each subtest has an increasing difficulty. For the present study, the following subtests the *LPS 3* and *LPS 7* were used. *LPS 3* measures logical reasoning, as an aspect of fluid intelligence. The aim is to indicate the incongruent element in each row of eight logically arranged symbols. The highest score to be achieved is 40. The respondent was given 5 minutes to complete the test. *LPS 7* requires mental rotation of letters in the plane – an ability attributed to fluid intelligence. The task consists of crossing out those symbols that are recognized as mirror images. The time limit for this subtest is 2 minutes. A maximum of 40 recognized symbols can be achieved. The number of correctly crossed-out symbols was used as a dependent variable.

### CFQ

The Cognitive Failures Questionnaire (CFQ; Broadbent et al., 1982) was used to assess performance in daily activities. *CFQ* is a scale including 26 questions related to attentional and memory lapses in the daily life, for example “Do you find you forget whether you have turned off a light or a fire or locked the door?,” or “Do you fail to listen to people’s names when you are meeting them?.” Frequent lapses are scored higher than less frequent ones and the total score is used as a dependent variable.

### Digit symbol test

The Digit Symbol Test (DST) measures aspects of focused attention and psychomotor speed (from the Nürnberg Age Inventory (NAI); Oswald and Fleischmann, 1986). In this test, the symbols on the test sheet have to be matched to the numbers 1 to 9 within 90 s. The maximum score is 93. The number of correct number and symbol assignments served as a dependent variable.

### Digit span

In the first part of the task, digit span forward (*DS forward,* from NAI; Oswald and Fleischmann, 1986), a series of digits with increasing length were orally given by the experimenter. The number of correctly memorized series of digits indicates short-term memory performance. Correct reproduction of two series with the same length indicates the correct response. The maximal performance is nine numbers.

In the second part of the task, digit span backward (*DS backward*) the presented number sequences must be repeated in reverse order. A maximum of eight consecutive numbers can be repeated. Two successive series of numbers with the same length indicate the correct response. This version of the task assesses working memory capacity. The sum of DS forward and backward reflects the general memory capacity (DS total) and was used as dependent variable.

### Stroop test

The color-word interference test (from NAI; Oswald and Fleischmann, 1986) consists of three tasks to test response inhibition to irrelevant stimuli. In the first task (*Stroop 1*) color words printed in blank ink are to be read as quickly as possible (e.g., “red,” “green”). The second task (*Stroop 2*) consists of naming the color bars. In the third task (*Stroop 3*) – the interference condition – the subjects are asked to name a series of the color words in which a color word is printed in colors which did not match the names of the colors (e.g., “GREEN” was printed in red). Here, word meaning, and print color are incongruent, with inhibition performance consisting of naming the color rather than reading the word as an automated response. The processing time of the interference condition as well as the difference between Stroop 3 and Stroop 2 (*Stroop 3–2*) are dependent variables.

### Verbal learning and memory test

The Verbal Learning and Memory Test (VLMT; Helmstaedter et al., 2001) is a translation of the Auditory Verbal Learning Test (AVLT; Rey, 1958). The VLMT measures the performance of verbal declarative episodic memory, which plays a major role in the performance of everyday tasks. Several memory subcomponents (learning efficacy, distractibility, delayed recall etc.) are measured by the test. The test material of the VLMT consists of a learning list and an interference list with 15 words each, which are semantically independent of each other. In addition, there is a recognition list with all words of the learning and interference list as well as 20 additional words. First, the subjects are presented with the learning list orally by the experimenter five times (VLMT 1 to VLMT 5). After each presentation, the words should be reproduced. This is followed by a single presentation of the interference list (VLMT I), which should be reproduced as well. Immediately after this, the participants are asked to reproduce the learning list presented at the beginning (VLMT 6). After about 30 min, which are used for the other tests, the learning list the subjects were asked to recall the original list (VLMT 7). Finally, the recall list, consisting of a total of 50 words, was presented orally (VLMT W). Here the task is to recognize the 15 words of the learning list.

Dependent variables were the total number of words from the learning list that the subject recalled in 5 trials (*VLMT Ʃ 1–5*), number of reproduced words from the interference list (*VLMT I*), number of words reproduced from the original list after interference (*VLMT 6*), number of words reproduced from the original list after 30 min (*VLMT 7*), loss of words reproduced from the original list after the interference list (trial 5 minus trial 6; *VLMT 5–6*), delayed free reproduction (trial 5 minus trial 7; *VLMT 5–7*) and the number of recognized words of the recognition list (minus errors; *VLMT W-F*).

### Trail making test

The Trail Making Test (TMT; Reitan, 1992) is a paper-pencil test consisting of two parts. Part A (TMT-A) measures processing speed and short-term memory by asking the participants to connect the numbers 1 through 25 consistently in ascending order.

In part B, the letters A to L and the numbers 1 to 13 are to be alternately connected in ascending order. In this dual task parallel processing of the two different subtasks “numbers” and “letters” is required. Part B (TMT-B) measures switch ability and divided attention. The difference between processing times for part B and A (TMT B-A) measuring components of executive functions was also included as a dependent variable in the analysis.

### Word fluency

The word fluency from LPS (Horn, 1983) measures cognitive flexibility and verbal speed. In this test, the participant is asked to find as many words as possible from the three given initial letters (F, K, R). For each initial letter the participant was given one minute. The total number of all words written down (*WF Written*) without repetition and rule violation was used as the test score. For the analog verbal version of the test (*WF Spoken*) the experimenter counted the number of spoken words beginning with the letters B, F, L. The total number of meaningful words was used as a dependent variable.

### *g*-factor

The outlined cognitive measures have been used to generate the *g*-factor also known as general intelligence. *g*-factor is a construct developed in psychometric studies of cognitive abilities and human intelligence. It is based on the observation that the performance of different cognitive tasks is positively correlated, reflecting the fact that an individual’s performance of one type of cognitive task tends to be comparable to that person’s performance of other cognitive tasks (Deary, 2020). The *g*-factor was assessed using structural equation modeling (SEM) with full information maximum likelihood (FIML), including the variables *d2, DST, LPS 3, LPS 7, MWT-B, WF Spoken, WF Written, VLMT Ʃ 1–5, VLMT W-F, DS forward, DS backward, Stroop 2, Stroop 3, TMT-A, TMT-B*. Individual regression analyses were calculated with age, sex, age^2^, age*sex, and age^2^*sex to extract the residuals. Then, the residuals were z-standardized (MW = 0, SD = 1). The result of the exploratory factor analysis indicated four factors: attention, verbal fluency, verbal memory and working memory, including the following tests: (a) Attention: *d2, Stroop 2, Stroop 3, TMT-A, TMT-B, DST, LPS 3, LPS 7*, (b) Verbal fluency: *WF Spoken, WF Written, MWT-B*, (c) Verbal memory: *VLMT Ʃ 1–5, VLMT W-F*, (d) Working memory: *DS forward, DS backward*.

**Figure 1:**
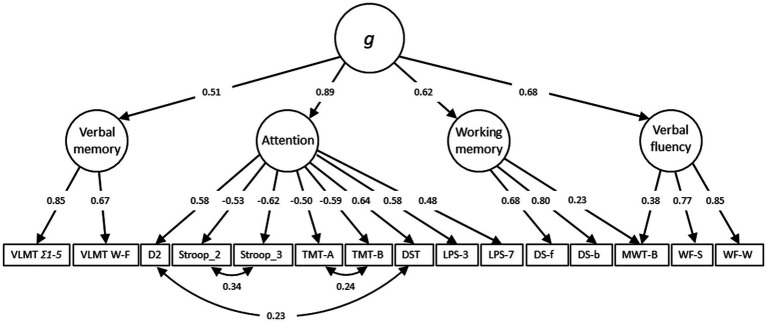
The factorial structure of the *g*-factor.

A second-order CFA (confirmatory factor analysis) yielded acceptable fit indices: χ2 (82) = 198.230, *p* < 0.001, root mean square error of approximation (RMSEA) = 0.049, comparative fit index (CFI) = 0.951, standardized root mean square residual (SRMR) = 0.041. Figure 1 illustrates the factorial structure of the *g*-factor.

### Data analysis

Descriptive statistics, frequencies, and distributions were calculated for all quantitative variables. A series of simple linear regression models were conducted. In each model, the independent variable (predictor) was the continuous variable of the relative power of the PWC-130 test assessing the level of PF, and the dependent variable was the continuous scores of the cognitive test. Missing data were handled with listwise deletion. The data was controlled for normality, linearity, and independence of residuals. Unstandardized and standardized *β,* standard error of the mean and the corresponding *t*-test, as well as the adjusted *R^2^* are reported. All statistical decisions were made at *p* < 0.05. No adjustment of the alpha level was applied to prevent type II error, i.e., not detecting possibly relevant associations. This is a common and consented strategy in epidemiologic and exploratory studies, where the information obtained by the data prevails over the number of tests (Rothman, 1990; Savitz and Olshan, 1995; Perneger, 1998; Bender and Lange, 2001). The reader can assess the strength of the associations for themselves using the provided statistical indices. Data were analyzed using the Statistical Package for Social Sciences (SPSS; IBM Statistics, Version 29).

The analysis was conducted in two steps: First, the relationship between PF and cognitive functions was tested in the entire sample of participants (20–70 years old) using the full range of variation in the data. To assess the modulation of the PF – cognition relationship by age, a continuous interaction term Age x PF was included in addition to the predictors Age and PF in the standard regression model. In the second step, the sample was dichotomized based on the 50^th^ percentile in younger to middle-aged group (YM; 20–45 years old; *M* = 32.3, SD = 7.2, n = 254; 64.2% female), and middle-aged to older group (MO; 46–70 years old; *M* = 56.4, SD = 6.6; n = 236; 57.6% female), relative to the median age of 46 years (See Footnote 1). The performance in the cognitive tests and subtests was analyzed in relation to PF level assessed by a quotient of maximum power in a bicycle ergometry test PWC-130 divided by body weight (W/kg). The median PF level in the YM group was 1.60 W/kg, and in the MO-group was 1.57 W/kg.

## Results

### Relationship between cognitive parameters and physical fitness in the whole sample

As evident from Table 1, PF was not associated with age as a continuous variable, but it was positively associated with subfactors constituting the *g*-factor: attention, verbal memory, verbal fluency, and working memory. Expectedly, PF was positively related to the reported weekly physical activity (*LPAQ*). General cognitive status measured by Mini Mental State Examination (*MMSE*) increased with the level of PF. Furthermore, working memory measured by digit span backward was positively predicted by PF. The same was true for a compound measure including digit span forward and backward (*DS-total*). Additionally, loss of words reproduced from the original list after the interference list in the VLMT (*VLMT 5–6*) was lower in higher level PF individuals. Similarly, loss of words in delayed free reproduction (*VLMT 5–7*) was lower in higher level PF. No further associations, particularly with specific executive tests such as Stroop, TMT, or word fluency tests were found.

**Table 1:** Results of the standard linear regression analyses for the prediction of age and PF on cognition in the entire sample.

Variable	*R^2^*	*b*	SE	β*	*t*	*p*
Age	0.001	−0.922	1.441	−0.031	−0.640	0.522
LPAQ: self-reported PF level	0.051	**97.618**	**20.763**	**0.225**	**4.702**	**<0.001**
Associations with cognitive factors based on SEM						
*g-*factor	0.010	**0.074**	**0.034**	**0.099**	**2.192**	**0.029**
Attention	0.013	**0.174**	**0.067**	**0.116**	**2.584**	**0.010**
Verbal Fluency	0.006	0.082	0.047	0.079	1.758	0.079
Verbal Memory	0.014	**0.145**	**0.056**	**0.117**	**2.601**	**0.010**
Working Memory	0.006	0.054	0.032	0.076	1.694	0.091
Associations with individual cognitive tests						
MMSE: Cognitive status	0.058	**0.687**	**0.133**	**0.246**	**5.179**	**<0.001**
d2: Sustained attention	0.002	3.187	3.952	0.040	0.806	0.420
MWT-B: Crystal. intelligence	0.001	0.194	0.313	0.030	0.620	0.535
DST: Psychomotor speed	0.000	0.471	1.245	0.019	0.379	0.705
LPS 3: Logical reasoning	0.003	0.658	0.576	0.056	1.144	0.253
LPS7: Mental rotation	0.001	−0.385	0.804	−0.023	−0.478	0.633
CFQ: Cognitive failures	0.006	−1.880	1.173	−0.078	−1.602	0.110
Digit span forward	0.005	0.294	0.210	0.069	1.400	0.162
Digit span backward	0.025	**0.706**	**0.214**	**0.160**	**3.295**	**0.001**
Digit span total	0.017	**1.000**	**0.375**	**0.130**	**2.664**	**0.008**
VLMT Ʃ 1–5: Learning span	0.002	0.912	0.974	0.046	0.937	0.350
VLMT I: Interference	0.005	0.328	0.224	0.072	1.464	0.144
VLMT 6: Post-interference	0.011	**0.631**	**0.290**	**0.106**	**2.176**	**0.030**
VLMT 7: Delayed recall	0.008	0.555	0.301	0.090	1.844	0.066
VLMT W-F: Delayed recognition	0.007	0.494	0.288	0.084	1.715	0.087
VLMT 5–6: Loss after interference	0.018	**−0.503**	**0.183**	**−0.134**	**−2.750**	**0.006**
VLMT 5–7: Loss after delay	0.012	**−0.427**	**0.194**	**−0.107**	**−2.202**	**0.028**
Stroop 1	0.001	−0.149	0.247	−0.030	−0.602	0.548
Stroop 2	0.000	0.112	0.348	0.016	0.323	0.746
Stroop 3	0.001	−0.437	0.729	−0.029	−0.599	0.549
Stroop 3–2: Interference	0.002	−0.550	0.614	−0.044	−0.895	0.371
TMT A	0.002	−0.831	0.941	−0.043	−0.882	0.378
TMT B	0.002	−2.481	2.687	−0.045	−0.924	0.356
TMT B-A: Cognitive flexibility	0.001	−1.651	2.329	−0.035	−0.709	0.479
Word fluency: Spoken	0.000	−0.287	1.343	−0.010	−0.213	0.831
Word fluency: Written	0.000	−0.022	0.823	−0.001	−0.027	0.979

*R*^2^ = *R*-quadrat (adjusted); *b* = unstandardized coefficients; SE = standard error, β* = standardized coefficients; *t = t-*value, *p =* value of *p*; SEM, Structural Equation Modeling. Bold values denote significant results.

In addition to the relation between age and PF shown in Table 1, the question whether age moderates the relationship between PF and cognition was explored in a follow-up analysis, in which an interaction term Age x PF was included in the regression models with Age and PF as predictors. The results of this analysis is presented in Table 2, showing that the interaction term was significant for the *g*-factor, verbal fluency, sustained attention (d2), psychomotor speed (DST), logical reasoning (LPS-3), and the Stroop interference test.

**Table 2:** Results of the interaction term age x PF using standard linear regression analyses for the prediction of age and PF on cognition in the entire sample.

Variable	*R^2^*	*b*	SE	β*	*t*	*p*
LPAQ: self-reported PF level	0.057	1.824	1.425	0.294	1.279	0.202
Associations with cognitive factors based on SEM						
*g-*factor	0.017	0.005	0.002	0.442	**2.107**	**0.036**
Attention	0.013	0.005	0.005	0.212	1.009	0.314
Verbal Fluency	0.017	0.008	0.003	0.493	**2.352**	**0.019**
Verbal Memory	0.012	0.005	0.004	0.259	1.232	0.218
Working Memory	0.004	0.003	0.002	0.235	1.115	0.266
Associations with individual cognitive tests						
MMSE: Cognitive status	0.092	0.009	0.009	0.211	1.045	0.296
d2: Sustained attention	0.229	0.611	0.221	0.515	**2.769**	**0.006**
MWT-B: Crystal. intelligence	0.189	0.001	0.019	0.005	0.028	0.978
DST: Psychomotor speed	0.330	0.150	0.071	0.382	**2.119**	**0.035**
LPS 3: Logical reasoning	0.214	0.081	0.036	0.445	**2.278**	**0.023**
LPS7: Mental rotation	0.075	0.078	0.053	0.309	1.455	0.146
CFQ: Cognitive failures	0.046	0.077	0.073	0.218	1.050	0.294
Digit span forward	0.019	0.002	0.014	0.024	1.115	0.908
Digit span backward	0.040	0.024	0.014	0.371	1.789	0.074
Digit span total	0.033	0.026	0.024	0.222	1.070	0.285
VLMT Ʃ 1–5: Learning span	0.223	−0.015	0.059	−0.050	−0.255	0.799
VLMT I: Interference	0.146	−0.027	0.014	−0.387	−1.899	0.058
VLMT 6: Post-interference	0.200	0.006	0.018	0.066	0.333	0.740
VLMT 7: Delayed recall	0.193	0.006	0.019	0.059	0.298	0.766
VLMT W-F: Delayed recognition	0.109	0.009	0.019	0.095	0.459	0.647
VLMT 5–6: Loss after interference	0.103	−0.009	0.013	−0.142	−0.678	0.498
VLMT 5–7: Loss after delay	0.108	−0.008	0.013	−0.135	−0.646	0.518
Stroop 1	0.078	−0.019	0.017	−0.249	−1.175	0.241
Stroop 2	0.021	−0.029	0.024	−0.263	−1.201	0.230
Stroop 3	0.221	−0.141	0.045	−0.611	**−3.129**	**0.002**
Stroop 3–2: Interference	0.208	−0.096	0.039	−0.490	**−2.490**	**0.013**
TMT A	0.177	−0.054	0.061	−0.178	−0.891	0.374
TMT B	0.173	−0.151	0.171	−0.178	−0.886	0.376
TMT B-A: Cognitive flexibility	0.099	−0.112	0.155	−0.153	−0.727	0.468
Word fluency: Spoken	0.006	0.003	0.093	0.006	0.029	0.977
Word fluency: Written	0.005	0.048	0.053	0.192	0.907	0.365

*R*^2^ = R-quadrat (adjusted); *b* = unstandardized coefficients; SE = standard error; β* = standardized coefficients; *t = t-*value, *p =* value of *p*; SEM, Structural Equation Modeling. Bold values denote significant results.

### Relationship between cognitive parameters and physical fitness in the YM group

Table 3 presents the linear relationship between the PF and cognitive variables in the YM group. Regarding subjectively reported level of PF a substantial positive relationship with PF assessed by PWC-130 was found. The regression model showed also that PF is a significant predictor of general cognitive status (*MMSE*). Additionally, PF predicted attentional and memory failures in daily life.

**Table 3:** Results of the standard linear regression analyses for the prediction of PF on cognition in the young to middle-aged (YM) group.

Variable	*R^2^*	*b*	SE	β*	*t*	*p*
LPAQ	0.043	73.339	23.768	0.207	**3.086**	**0.002**
Associations with cognitive factors based on SEM						
*g-*factor	0.000	−0.001	0.051	−0.001	−0.019	0.985
Attention	0.003	0.081	0.099	0.056	0.817	0.415
Verbal Fluency	0.001	−0.026	0.070	−0.026	−0.376	0.707
Verbal Memory	0.003	0.075	0.090	0.058	0.840	0.402
Working Memory	0.000	0.004	0.051	0.005	0.071	0.943
Associations with individual cognitive tests						
MMSE: Cognitive status	0.035	0.429	0.156	0.186	**2.755**	**0.006**
d2: Sustained attention	0.004	−5.164	5.490	−0.064	−0.941	0.348
MWT-B: Crystal. intelligence	0.003	0.361	0.443	0.056	0.814	0.417
DST: Psychomotor speed	0.010	−2.270	1.537	−0.101	−1.477	0.141
LPS 3: Logical reasoning	0.003	−0.623	0.735	−0.058	−0.848	0.398
LPS7: Mental rotation	0.007	−0.623	0.735	−0.058	−0.848	0.398
CFQ: Cognitive failures	0.025	−3.902	1.658	−0.160	**−2.353**	**0.020**
Digit span forward	0.001	0.177	0.313	0.039	0.564	0.574
Digit span backward	0.007	0.381	0.308	0.085	1.237	0.218
Digit span total	0.005	0.557	0.551	0.069	1.011	0.313
VLMT Ʃ 1–5: Learning span	0.000	−0.016	1.117	−0.001	−0.015	0.988
VLMT I: Interference	0.013	0.549	0.330	0.114	1.665	0.097
VLMT 6: Post-interference	0.001	0.173	0.325	0.036	0.531	0.596
VLMT 7: Delayed recall	0.001	0.174	0.351	0.034	0.495	0.621
VLMT W-F: Delayed recognition	0.002	0.199	0.323	0.042	0.616	0.539
VLMT 5–6: Loss after interference	0.002	−0.266	0.214	−0.085	−1.242	0.216
VLMT 5–7: Loss after delay	0.002	−0.267	0.238	−0.077	−1.121	0.264
Stroop 1	0.001	0.122	0.348	0.024	0.350	0.727
Stroop 2	0.004	0.417	0.483	0.059	0.865	0.388
Stroop 3	0.006	0.973	0.892	0.075	1.091	0.276
Stroop 3–2: Interference	0.003	0.555	0.682	0.056	0.814	0.417
TMT A	0.001	0.457	1.097	0.029	0.416	0.678
TMT B	0.001	1.414	2.686	0.036	0.526	0.599
TMT B-A: Cognitive flexibility	0.001	0.957	2.302	0.029	0.416	0.678
Word fluency: Spoken	0.000	−0.529	1.860	−0.020	−0.284	0.776
Word fluency: Written	0.002	−0.727	1.188	−0.042	−0.612	0.541

R^2^ = *R*-quadrat (adjusted); *b* = unstandardized coefficients; SE = standard error; β* = standardized coefficients; *t = t-*value, *p* = value of *p*; SEM, Structural Equation Modeling. Bold values denote significant results.

### Relationship between cognitive parameters and physical fitness in the MO group

The regression models for the MO group are presented in Table 4. Similar to YM group, the MO group showed a significant positive relationship between subjectively reported PF level and PF measured by the PWC-130. The regression model showed that PF was a significant predictor of general cognitive status (*MMSE*). PF in MO group substantially predicted several cognitive parameters: Attention, Verbal Fluency and Memory factors constituting the *g*-factor which was also predicted by PF. In addition, PF was positively related to attentional endurance measured by the d2 test, digit span backward, digit span total score, logical reasoning (LPS 3), less loss of words reproduced from the original list after the interference list (trial 5 minus trial 6) and delayed free reproduction (trial 7). Finally, PF was associated with less time to perform the Stroop interference list and in difference between the interreference list and the control list.

**Table 4:** Results of the standard linear regression analyses for the prediction of PF on cognition in the middle-aged to older (MO) group.

Variable	*R^2^*	*b*	*SE*	β*	*t*	*p*
LPAQ	0.062	122.99	33.832	0.249	**3.635**	**<0.001**
Associations with cognitive factors based on SEM						
*g-*factor	0.024	0.245	0.102	0.155	**2.402**	**0.017**
Attention	0.021	0.154	0.068	0.147	**2.261**	**0.025**
Verbal Fluency	0.034	0.222	0.077	0.186	**2.884**	**0.004**
Verbal Memory	0.012	0.072	0.044	0.107	1.648	0.101
Working Memory	0.024	0.245	0.102	0.155	**2.402**	**0.017**
Associations with individual cognitive tests						
MMSE: Cognitive status	0.091	0.922	0.206	0.302	**4.480**	**<0.001**
d2: Sustained attention	0.026	10.393	4.485	0.162	**2.317**	**0.021**
MWT-B: Crystal. intelligence	0.001	0.175	0.377	0.033	0.464	0.643
DST: Psychomotor speed	0.018	2.816	1.468	0.134	1.918	0.057
LPS 3: Logical reasoning	0.026	1.741	0.751	0.162	**2.318**	**0.021**
LPS7: Mental rotation	0.001	0.536	1.051	0.036	0.510	0.611
CFQ: Cognitive failures	0.001	−0.607	1.530	−0.028	−0.396	0.692
Digit span forward	0.010	0.394	0.276	0.100	1.426	0.155
Digit span backward	0.058	1.020	0.291	0.241	**3.505**	**<0.001**
Digit span total	0.039	1.414	0.496	0.198	**2.849**	**0.005**
VLMT Ʃ 1–5: Learning span	0.007	1.666	1.369	0.086	1.216	0.225
VLMT I: Interference	0.001	0.094	0.273	0.024	0.344	0.731
VLMT 6: Post-interference	0.031	1.051	0.419	0.175	**2.510**	**0.013**
VLMT 7: Delayed recall	0.022	0.902	0.428	0.147	**2.108**	**0.036**
VLMT W-F: Delayed recognition	0.015	0.784	0.447	0.123	1.754	0.081
VLMT 5–6: Loss after interference	0.031	−0.711	0.280	−0.177	**−2.539**	**0.012**
VLMT 5–7: Loss after delay	0.019	−0.563	0.290	−0.136	−1.942	0.054
Stroop 1	0.009	−0.440	0.320	−0.097	−1.376	0.170
Stroop 2	0.001	−0.201	0.493	−0.029	−0.409	0.683
Stroop 3	0.029	−2.409	0.953	−0.171	**−2.529**	**0.012**
Stroop 3–2: Interference	0.019	−1.762	0.877	−0.137	**−2.009**	**0.046**
TMT A	0.008	−1.800	1.392	−0.091	−1.293	0.197
TMT B	0.009	−5.790	4.246	−0.096	−1.364	0.174
TMT B-A: Cognitive flexibility	0.005	−3.990	3.847	−0.073	−1.037	0.301
Word fluency: Spoken	0.000	0.043	1.954	0.002	0.022	0.983
Word fluency: Written	0.002	0.693	1.127	0.043	0.615	0.539

*R*^2^ = R-quadrat (adjusted); *b* = unstandardized coefficients; SE = standard error; β* = standardized coefficients; *t = t-*value; *p =* value of *p*; SEM, Structural Equation Modeling. Bold values denote significant results.

## Discussion

The present study aimed to investigate whether physical fitness is related to the same or different cognitive functions in younger to middle-aged and middle-aged to older adults. To this end, an objectively measured level of physical fitness assessed by bicycle ergometry was related to specific cognitive functions using a series of standardized psychometric tests. Regression models showed that PF was positive related to general cognitive performance as a proxy of general intelligence (*g*-factor) and its subcomponents such as visual attention and verbal memory in the entire sample. A subsequent regression model including an interaction term Age x PF showed that this association was moderated by age and that age also moderates the relationship between PF and individual cognitive domains. In a subsequent step, the sample was split in two age groups (YM and OM). In the YM group, higher PF levels predicted only MMSE, and a lower risk for attentional and memory lapses leading to cognitive failures in daily life (CFQ), whereas other associations were far from being significant. In contrast, consistent associations between PF and cognitive function were observed in the MO group. Apart from a clear relationship between PF and MMSE, higher PF levels were associated with improved performance on sustained attention in the attentional endurance test (d2), digit span backward, measuring working memory capacity, reasoning (LPS 3), various aspects of verbal memory (VLMT; reproduction of a learning list after interference, delayed reproduction), and lower Stroop interference. In addition, the results showed that PF predicted visual attention, verbal fluency and working memory as subdimensions of the *g*-factor, as well as the *g*-factor itself. This supports the hypothesis that PF is positively related to general intelligence, at least in middle to older age.

The results are consistent with previous findings showing higher performance with high-level PF in executive function tasks such as working memory (Colcombe and Kramer, 2003; Voelcker-Rehage et al., 2010), Stroop interference task (Liu-Ambrose, 2010; Gajewski and Falkenstein, 2015a; Coetsee and Terblanche, 2017), as well as in general cognition in older adults (Laurin et al., 2001; Weuve et al., 2004; Middleton, 2011; Iso-Markku et al., 2016). Association between PF and MMSE in young to middle-aged healthy adults has not been previously reported, as the MMSE is intended as an instrument to assess mild cognitive impairment as an early stage of dementia. However, the present study is a part of a longitudinal study, and such a standardized screening instrument was included to evaluate changes in cognitive status across decades.

Another surprising finding was the substantially lower risk of attentional and memory lapses and cognitive errors assessed with the CFQ in the YM group, with no other effects in the neuropsychological tests, suggesting that higher PF might reduce forgetfulness, distractibility, and thinking blunders. However, as recently reported (Goodman et al., 2022), CFQ scores did not predict performance on objective neuropsychological tests but were related to a range of psychological stress symptoms, as originally suggested by Broadbent et al. (1982). This suggests that a high CFQ score in physically fit young to middle-aged adults is related to increased stress resilience rather than specific cognitive functions.

The main question of the study was related to the differences regarding the responsivity of specific cognitive functions to PF across the adult lifespan and specifically in YM and MO adults. The analyses showed that the relationship between PF and cognition was modulated by age in respect to general intelligence and some of the specific cognitive functions such as sustained attention, psychomotor speed, logical reasoning, or interference processing but not in other domains such as verbal memory. In general, we observed that PF was associated with substantial and greater gains in cognitive function in the MO group compared with YM group, who showed virtually no association between neuropsychological tests and PF levels. The only exception was the mentioned MMSE (with a stronger association in the MO than the YM group), suggesting that the screening tool is sensitive to detect already small differences in cognitive status independently on age. Indeed, the substantial association between PF and MMSE in the YM group is unexpected as one would expect a ceiling effect. MMSE was used in the present study to monitor changes and potential onset of MCI or other types of dementia over the planned 20-year study period. It includes questions regarding spatial–temporal orientation, attention, arithmetic, comprehension, and working memory reflecting crucial cognitive functions that can be positively affected by physical activity and enhanced brain metabolism or inversely some slight impairments of brain metabolism may be expressed in lower MMSE scores.

Overall, no final conclusion can be drawn about the association between PF and specific cognitive functions in the YM group as the relationship was hardly observed in this age group compared to their older counterparts (Middleton et al., 2010; Voelcker-Rehage and Niemann, 2013; Cox et al., 2016, for reviews). Physical activity at a young age, particularly as a teenager, has been shown to positively impact cognitive performance in later life (Middleton et al., 2010), but the positive relationship between PF and cognition is stronger in middle-aged to old adults than in young to middle-aged adults (Hayes et al., 2014; Barnes, 2015; Wade et al., 2020; Rieker et al., 2023). Moreover, available studies reporting brain function, connectivity, and structure related to different types of physical activity in the young to middle-aged groups are underrepresented compared with studies with older adults (see Figure 1 in Voelcker-Rehage and Niemann, 2013; Erickson et al., 2019; Stillman et al., 2020). The lack of, or weak positive association between PF and cognition in the young and middle-aged adult group may have led to this publication bias. Overall, studies show that the benefits of physical activity on cognition tend to increase with age (Stones and Kozma, 1988; Newson and Kemps, 2006). This may be a reason for the few and often inconsistent results in young and middle-aged adults (Voelcker-Rehage and Niemann, 2013; Cox et al., 2016; Diamond and Ling, 2016; Erickson et al., 2019; Wade et al., 2020; Kachouri et al., 2022).

This age difference seems to be due to neurobiological reasons: The crucial aspect of PF on brain activity and structure is improved oxygen uptake in the blood. Since blood oxygenation is optimal in young and middle-aged people, physical activity does not additionally contribute to the improvement of brain function and cognition. In this case, PF does not have serious consequences for cognitive performance, and both seem to be independent. In other words, healthy young adults with low and high levels of PF did not differ in their cardiovascular capacity (i.e., maximal oxygen uptake) and thus in their cognitive abilities. In contrast, older individuals have a higher risk of cardiovascular decline and lower brain oxygen saturation, which can be significantly improved by PF (Polk et al., 2022). Second, PF and improved oxygenated blood perfusion affect cognition (Schapkin et al., 2012) by influencing molecular events related to the control of energy metabolism and synaptic plasticity (Gomez-Pinilla and Hillman, 2013) by causing an increase in proteins such as nerve growth factor, brain-derived neurotrophic factor (BDNF) (Kirk-Sanchez and McGough, 2013; de Assis and Almondes, 2017) or insulin-like growth factor type 1 (IGF-1), which stimulate neuronal development and prevent death of neurons (Kramer and Erickson, 2007; Stillman et al., 2020). Specifically, neuronal plasticity promotes neurogenesis and synaptic plasticity in the hippocampus and frontal brain areas associated with memory and executive functions (Gomez-Pinilla and Hillman, 2013). The functional mechanisms underlying the benefits of PF have additionally been linked to increased brain volume, increased functional connectivity between brain areas, and cortical thickness (Voelcker-Rehage and Niemann, 2013; Brehmer et al., 2014; Stillman et al., 2020). Thus, it can be assumed that these complex mechanisms are compromised in older age but can be re-activated by cardiovascular adaptation induced by physical activity. The lower intraindividual variability of brain function in physically fit older adults and the increased interindividual variability between low and high PF in old age (Gajewski and Falkenstein, 2015a) might lead to a stronger positive association between PF and cognitive performance than in young to middle-aged adults.

## Limitations and future directions

The present study is a cross-sectional analysis of data from the *Dortmund Vital Study*, whereas a longitudinal approach is a more conclusive way. In particular, changes in PF and cognitive functioning can be tracked over decades in the same sample. Therefore, the weak association between PF and cognitive functions in younger to middle-aged group should become stronger as participants age. This would allow to substantiate the results and to draw causal conclusions about the effects of PF on specific cognitive functions across the lifespan when the longitudinal data of the *Dortmund Vital Study* become available in a few years.

Second, we did not consider in the analyses potentially confounding variables such as gender, education, sleep quality, sedentary behavior, mental health, nutrition, immunological status, type of work, social contacts, personality, quality of life, etc. (c.f., Gajewski et al., 2023) that might moderate the association between PF and cognition. One reason for this is that missing data were handled with listwise deletion, which means that missing values in any of these variables reduce the total number of cases and reduce the final sample and power. Nonetheless, these variables will be collected during follow-up and will be included as control variables in the longitudinal analysis of the data. For this reason, advanced statistical approaches such as Generalized Estimating Equation or Mixed Effect Regressions will be used to handle with missing data in the longitudinal design.

Relevant strengths of the study include the fact that the participants were not informed about the specific questions of the present study and that test selection in this context was blind. Finally, selection bias can be largely excluded, as this study is a sample that does not differ from the general population in critical parameters, as described in the study protocol of the *Dortmund Vital Study*.

## Conclusion

The present study showed a positive relationship between physical fitness and cognitive functioning. However, this benefit was driven by a substantial association between PF and cognition in middle-aged to older adults, whereas only a weak association between PF and cognition was found in younger to middle-aged adults. In the middle-aged to older group, the association was stronger and concerned a variety of cognitive functions, such as attention, working memory and executive functions. Additionally, there was a general relationship between the *g*-factor as a proxy of fluid intelligence and PF which was moderated by age. The findings should be replicated in a longitudinal design to draw causal conclusions about the changes of the PF – cognition relationship across the lifespan.

## Data availability statement

The study data are available upon reasonable request from the authors [see the Research Data Management section in the study protocol by Gajewski et al. (2022)].

## Ethics statement

The studies involving human participants were reviewed and approved by Ethics Committee of IfADo (A93-1). The participants provided their written informed consent to participate in this study.

## Author contributions

PG designed the study, monitored data collection, analyzed data, and drafted the manuscript. SG designed the study, monitored data collection, and drafted the manuscript. JD and EG assisted in statistical analyses. KG, TK, and JH are responsible for the cardiovascular parameters and the corresponding data analysis. EW is the principal investigator of the study, designed the study, and supervised the organization of the measures. All authors revised and approved the manuscript.

## Funding

The *Dortmund Vital Study* is funded by the institute’s budget. Thus, the study design, collection, management, analysis, interpretation of data, writing of the report, and the decision to submit the report for publication is not influenced or biased by any sponsor.

## Conflict of interest

The authors declare that the research was conducted in the absence of any commercial or financial relationships that could be construed as a potential conflict of interest.

## Publisher’s note

All claims expressed in this article are solely those of the authors and do not necessarily represent those of their affiliated organizations, or those of the publisher, the editors and the reviewers. Any product that may be evaluated in this article, or claim that may be made by its manufacturer, is not guaranteed or endorsed by the publisher.

## Footnotes

^1^We use the terms YM and MO exclusively to describe age groups below and above the median age. These terms do not necessarily correspond to the terms used in the literature for age groups.
